# Phonological memory in sign language relies on the visuomotor neural system outside the left hemisphere language network

**DOI:** 10.1371/journal.pone.0177599

**Published:** 2017-09-20

**Authors:** Yuji Kanazawa, Kimihiro Nakamura, Toru Ishii, Toshihiko Aso, Hiroshi Yamazaki, Koichi Omori

**Affiliations:** 1 Human Brain Research Center, Kyoto University Graduate School of Medicine, Kyoto, Japan; 2 Department of Otolaryngology-Head and Neck Surgery, Kyoto University Graduate School of Medicine, Kyoto, Japan; 3 Faculty of Human Sciences, University of Tsukuba, Tsukuba, Japan; Weill Cornell Medical College, UNITED STATES

## Abstract

Sign language is an essential medium for everyday social interaction for deaf people and plays a critical role in verbal learning. In particular, language development in those people should heavily rely on the verbal short-term memory (STM) via sign language. Most previous studies compared neural activations during signed language processing in deaf signers and those during spoken language processing in hearing speakers. For sign language users, it thus remains unclear how visuospatial inputs are converted into the verbal STM operating in the left-hemisphere language network. Using functional magnetic resonance imaging, the present study investigated neural activation while bilinguals of spoken and signed language were engaged in a sequence memory span task. On each trial, participants viewed a nonsense syllable sequence presented either as written letters or as fingerspelling (4–7 syllables in length) and then held the syllable sequence for 12 s. Behavioral analysis revealed that participants relied on phonological memory while holding verbal information regardless of the type of input modality. At the neural level, this maintenance stage broadly activated the left-hemisphere language network, including the inferior frontal gyrus, supplementary motor area, superior temporal gyrus and inferior parietal lobule, for both letter and fingerspelling conditions. Interestingly, while most participants reported that they relied on phonological memory during maintenance, direct comparisons between letters and fingers revealed strikingly different patterns of neural activation during the same period. Namely, the effortful maintenance of fingerspelling inputs relative to letter inputs activated the left superior parietal lobule and dorsal premotor area, i.e., brain regions known to play a role in visuomotor analysis of hand/arm movements. These findings suggest that the dorsal visuomotor neural system subserves verbal learning via sign language by relaying gestural inputs to the classical left-hemisphere language network.

## Introduction

Sign language is a visuospatial human language with sign-based codes (e.g., handshape, location and movement) distinct from spoken sounds and has its own grammatical structures to convey linguistic signals comparable to spoken language [1]. For deaf people, sign language is not only an everyday communication tool but also serves as an essential medium for verbal learning, including vocabulary acquisition [2]. For spoken language, such verbal learning is thought to heavily rely on the phonological short-term memory (STM), which is known to play a key role in the acquisition process of vocabulary knowledge [3]. In fact, the neural substrate of the phonological STM is also known to largely overlap the left frontotemporal cortex involved in spoken language processing [4]. By contrast, it remains open whether the verbal STM via signed language is mediated by the same left hemisphere network for spoken language. Rather, some additional neural systems for visuomotor analysis of upper limb movements may play a role in verbal learning via sign language.

Indeed, functional neuroimaging data show that understanding of signed language relies not only on the classical left-hemisphere network for spoken language, including the left inferior frontal gyrus and superior temporal sulcus, but also on other neural components involved in non-linguistic gestures [5]. On the other hand, unlike the visual recognition of gestural inputs, verbal learning via sign language is shown to rely on the shared left-hemisphere language network which does not comprise the dorsal parietal cortex [6]. This seems plausible because (1) proficient signers may rely on the same ventral object-processing stream in the visual recognition of fingerspelling and other linguistic stimuli (i.e., written letters) and (2) verbal STM in itself is thought to buffer a higher-order, abstract level of linguistic representations independent of sensory input modality. However, other brain imaging studies with deaf and hearing signers suggest that brain systems for verbal STM also include two distinct neural components, one in the left-lateralized frontoparietal network shared with spoken language processing and additional modality-specific components in dorsal parietal cortex [7, 8]. Given those apparently conflicting results, it remains unclear whether and to what extent the dorsal parietal region contributes to the verbal STM via sign language.

Here it is important to note that most those previous studies used variously different experimental settings with hearing and deaf participants, thereby creating a large confound in comparing the neurocognitive basis of verbal STM between spoken and signed languages. That is, radically different types of stimuli and tasks (e.g., signed stimuli for deaf signers and auditory stimuli for hearing speakers) have been used to compare neural correlates of verbal STM between deaf signers and hearing speakers. Obviously, however, any neural activation differences obtained from such comparisons are likely to arise from variously different factors, such as the effects of stimuli, task and differences in language development and socioeconomic status between hearing speakers and deaf signers.

In the present study, we used event-related functional magnetic resonance imaging (fMRI) to identify the modality-independent network common to the two language systems and the putative, specific neural components for sign language. We compared neural activation between letter and fingerspelling stimuli while sign interpreters (i.e., proficient bilinguals of both language modalities) were engaged in a sequence memory span task. Here, fingerspelling has one-to-one correspondence with written letters and is thought to mediate phonological STM in sign language [9]. Specifically, the present study focused on neural activation during the maintenance stage of verbal information following visual stimulus encoding, thereby isolating the neural basis of the modality-independent and abstract level of verbal STM beyond the differences in sensory-input modality.

## Material and methods

### Participants

Thirteen healthy volunteers (five males, age-range 23–53 years, mean 43.3 years) participated in the present study. All of them were right-handed, normal hearing native Japanese speakers born to and raised by normal hearing parents. All were professional Japanese sign interpreters who had started learning sign language in adulthood. The mean age of acquisition was 21.0 years (range 17–29 years), whereas the mean duration of sign language use was 22.4 years (range 19–35 years). None of them had known neurological or psychiatric disorders. All had normal or corrected-to-normal vision and gave written informed consent prior to the experiment. The protocol of this study was approved by the ethical committee of Kyoto University Hospital (e-mail: ethcom@kuhp.kyoto-u.ac.jp).

### Behavioral tasks

Following experimental procedures previously used for working memory research [10, 11], we constructed 56 nonsense syllable sequences (4–7 syllables in length) by combining low-frequency bi-syllabic words selected from a standard lexical database of the Japanese language [12]. We extracted the frequency of each bi-syllabic word occurrence from the lexical database and calculated the overall frequency of each sequence as phonotactic frequency [13]. The phonotactic frequency (number of occurrences in the corpus) was matched between letter and finger stimuli at each level of sequence length as followed; 1082±639 (4 syllables), 1860±691 (5 syllables), 1884±610 (6 syllables), 2538±1092 (7 syllables) in letter materials and 1010±445 (4 syllables), 1905±694 (5 syllables), 1970±858 (6 syllables), 2444±1008 (7 syllables) in finger materials. For each syllable length, there were 14 stimulus pairs, including seven pairs which were identical to each other (e.g., he-mi-ro-ne-yu and he-mi-ro-ne-yu) and seven pairs which differed from each other by only one vowel or consonant (e.g., mu-he-k*e*-nu-a and mu-he-k*o*-nu-a).

**Fig 1:**
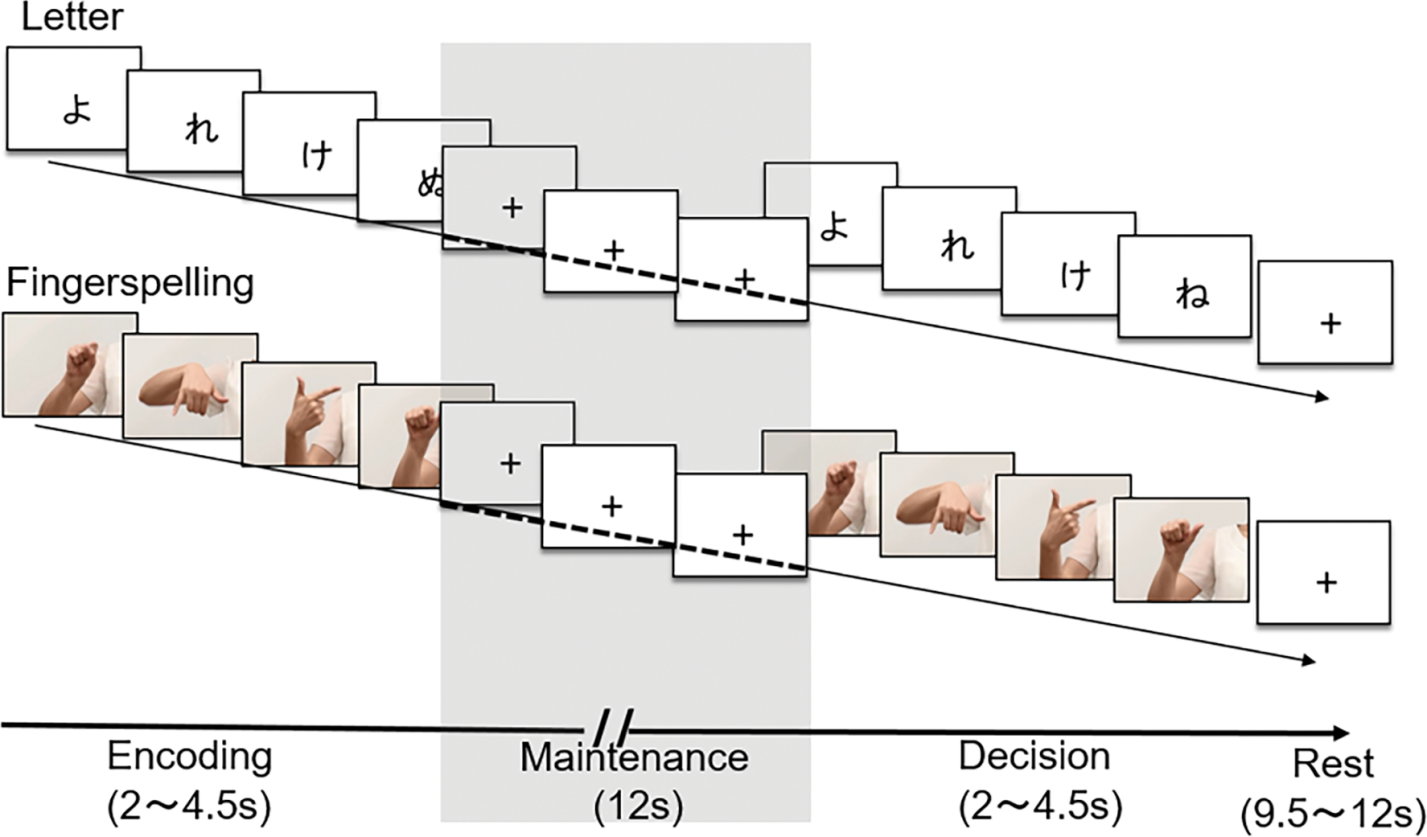
Experimental design of the sequence memory span task. On each trial, a nonsense sequence of 4–7 syllables was presented either as letters (Japanese kana) or as fingerspelling. Participants were instructed to read and memorize the first syllable sequence during encoding stage and keep the syllable sequence for 12 seconds during maintenance stage (highlighted). They then viewed another syllable sequence and decided by key-press whether or not two sequences were identical during the decision phase.

A sequence memory span task was created using printed letters (Japanese kana script) and moving images of fingerspelling. We recorded fingerspelling movements produced by a deaf female signer and displayed them as fingerspelling stimuli. Each syllable was serially presented at the rate of 500 ms and 650 ms for the letter and finger conditions, respectively, since longer presentation time is generally needed for signs than for written words [1]. Each trial comprised three stages, i.e., encoding, maintenance and decision (Fig 1). On each trial, participants were instructed to memorize a sequence of 4–7 syllables, presented either as letters or as fingerspelling, during the encoding stage lasting 2–5 s. After all visual stimuli were presented and cleared from the screen, participants were requested to covertly hold the syllable sequence during the maintenance stage for 12 s. At the subsequent decision stage, they were presented with another syllable sequence which could be identical to or slightly different from the one presented during encoding stage. Participants decided by button-press whether the pair of syllable sequences were the same or different. The next trial started following an inter-trial interval of either 9.5 or 12 s.

Each participant received seven sessions, each consisting of 16 trials (~eight trials for each of the letter and finger conditions). Each session included two trials for each level of sequences (4 to 7 syllables) for each of the letter and finger conditions. The order of sequence length and stimulus type were randomized within participants. Visual stimuli were presented with E-prime 2.0 software (Psychology Software Tools, Pittsburgh 2000). Prior to MRI scanning (see below), each participant performed practice trials with dummy stimulus sequences outside and inside the scanner. For each participant, we calculated accuracy rate for each sequence length (4–7 syllables) for each of the letter and finger conditions. These accuracy rates were evaluated by the use of analysis of covariance (ANCOVA) with a factor of stimulus type (letter and finger) and a covariate of sequence length (4–7 syllables).

We further devised a self-rating scale to assess a possible variability in cognitive strategy which participants could adopt during the maintenance stage. Previous research suggests that verbal working memory system involves two different cognitive mechanisms, i.e., phonological and visuospatial systems [14]. According to this framework, one effective strategy for maintenance is “phonological”, whereby participants covertly articulate speech in mind through translating syllable sequences into a phonological format. The other is “visuospatial”, in which participants covertly reactivate a visuospatial memory of stimulus sequence displayed on the screen. These components may contribute to the maintenance stage differently between the letter and finger conditions, since letters are likely to activate the phonological system more greatly than fingers, whereas fingers probably involve the visuospatial component more greatly than letters.

Immediately after MRI scanning, participants were asked to report their subjective reliance on these two strategies while holding the sequence memory for each sequence length for each condition. In scoring each strategy, a 5-point scale was assigned to the degree of dependence (0 = not at all, 1 = a little, 2 = often, 3 = very often, 4 = all the time). We calculated the differences in the score between phonological and visuospatial strategies for each subject (‘phonological’-‘visuospatial’) to assess the degree of dependency on phonological strategy compared to visuospatial strategy. These scores were then evaluated by the use of ANCOVA with a factor of stimulus type (Letter and Finger) and a covariate of sequence length (4–7 syllables).

### Imaging procedure

Imaging data were acquired using a Siemens Trio 3 T head scanner with 32 channel phased-array head coil. The echo planer imaging (EPI) data were acquired using recently developed multiband EPI sequence [15]: TR = 1.0 s, TE = 30 ms, flip angle = 60°, field of view (FOV) = 192 mm × 192 mm, multiband acceleration factor = 3, voxel size 3 × 3 × 3 mm, 48 axial slices. The multiband EPI parameters were 3- dimensional T1-weighted images with MPRAGE sequence were acquired with following parameters: TR = 2.0 s, TE = 3.37 ms, inversion time = 990 ms, FOV = 256 mm, voxel size = 1 × 1 × 1 mm, flip angle = 8°, 130 Hz bandwidth. Each participant received seven scanning sessions, each lasting 492 s.

### Imaging data analysis

Imaging data were preprocessed and analyzed using the Statistical Parametric Mapping (SPM8; Wellcome Department of Cognitive Neurology, London, UK). Functional Images were corrected for head movement, normalized to the standard brain space by the Montreal Neurological Institute (MNI) space using T1 image unified segmentation (the resampling voxel size was 2 × 2 × 2 mm), and smoothed with a 6-mm Gaussian kernel.

For the first-level analysis, we constructed a general linear model for assessing functional images with a 2 × 4 × 3 factorial design: stimulus type (letter or finger), sequence length (4–7 syllables) and stages (encoding, maintenance and decision). High-pass temporal filtering (128 Hz) was applied to the fMRI time-series data. For each participant, eight contrast images specific to the maintenance stage were calculated relative to the baseline condition by convolving known time-series of trials with a canonical hemodynamic response function and its time derivative. For each of the encoding and decision stages, we also created a contrast image (collapsed across the four levels of syllable length) per condition (i.e., letters and fingers) per participant. Here, decision making was assumed to occur when participants made behavioral response by key-press. Scanning sessions whose behavioral accuracy was less than 70% were excluded from further analysis.

For the second-level analysis, we submitted the eight contrast images for the maintenance stage per participant to one-way ANOVA to examine the effects of stimulus modality and sequence length on brain activation. We conducted a conjunction analysis to delineate modality independent brain areas for verbal STM by isolating voxels commonly activated by the letter and finger conditions during this maintenance stage. For each of the encoding and decision stages, we also submitted a contrast image per participant (see above) to one-way ANOVA to examine the effects of stimulus modality on brain activation during the encoding and decision stages. We further performed psychophysiological interaction (PPI) analysis to search for brain regions showing stimulus-dependent changes in functional connectivity with the left IFG previously associated with lexical learning [16]. In brief, PPI treats neural coupling of one area to another affected by experimental or psychological context [17]. Regional responses per session per participant were extracted by calculating the principal eigenvariate across all voxel within a 5-mm sphere centered at the most significant voxel of the left IFG during the maintenance stage derived from the group analysis. We then computed the PPI regressor as an element-by-element product of the IFG activity and a vector coding for the differential effect of letter and finger condition (1 for letter condition, -1 for finger condition) per session per participant. Two contrast images corresponding to ‘letter > finger condition’ and ‘finger > letter condition’ were created per participant and submitted to a second-level analysis (one-sample t test) to examine whether across-participant means differed from zero for each contrast. By selecting those voxels showing the between-conditions differences (letter vs. finger) as described above (inclusive masking with voxel-level p<0.001, uncorrected), brain regions showing significant shifts of functional connectivity with stimulus type were identified. Unless stated otherwise, statistical significance was examined with p < 0.05, corrected for multiple comparisons at the voxel level over the whole brain (family-wise error), with a cluster size threshold > 20 voxels. Activated brain regions were identified according to a probabilistic atlas [18]. All behavioral and MRI datasets analyzed in the present study have been deposited in the OpenfMRI database (https://openfmri.org/dataset/ds000237).

## Results

### Behavioral data

Mean accuracy for the memory span task was 82.9% for the letter condition and 80.4% for the finger condition, respectively (see Table 1 for the accuracy data for each condition). ANCOVA revealed a significant effect of sequence length (F [1, 12] = 96.5, p < 0.0001). The effect of stimulus type (F [1, 12] = 1.7, p = 0.2) and the interaction between stimulus type and sequence length were both non-significant (F [3, 12] = 0.7, p = 0.4). This finding suggests that the level of accuracy decreased with sequence length while the magnitude of accuracy reduction did not differ between letters and fingers.

**Table 1:** Mean accuracy (SD) for the letter and fingerspelling conditions during fMRI.

Sequence length	Stimulus modality	
	*Letter*	*Fingerspelling*
4	87.9 (7.9)	92.9 (9.2)
5	93.4 (8.5)	84.6 (12.0)
6	80.8 (11.4)	74.2 (9.5)
7	70.3 (8.7)	72.0 (13.8)
Total	82.9 (12.5)	80.4 (13.6)

**Fig 2:**
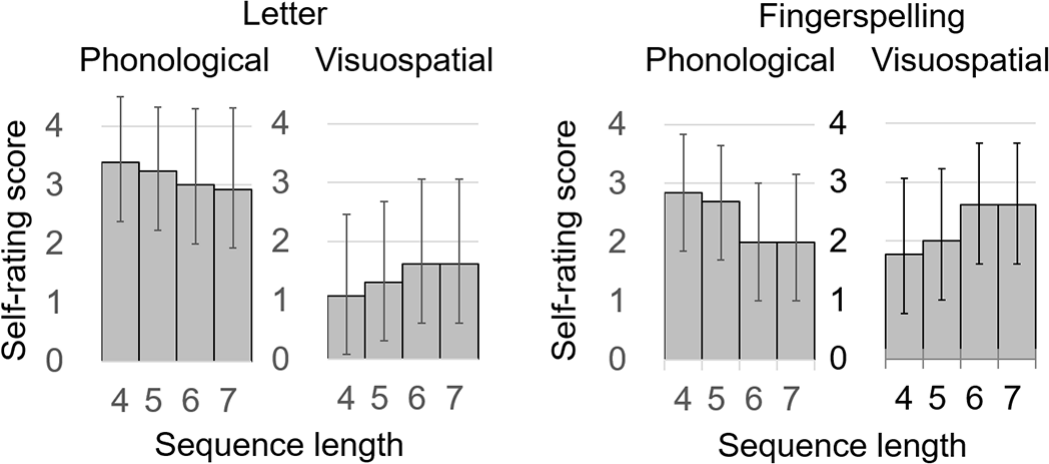
Self-rating scores of cognitive strategies used during the maintenance stage. ‘Phonological’ represents the degree of subjective reliance on phonological imagery of spoken sounds, whereas ‘Visuospatial’ on visuospatial imagery of letter- or finger-shapes (0 = not at all, 4 = all the time). After all imaging sessions were completed, participants were requested to recall these covert strategies that they had used during maintenance. Irrespective of the stimulus type (i.e. letters or fingers), participants relied on the phonological memory more greatly than on the visuospatial memory. However, this subjective reliance on phonological memory did not significantly differ between letters and fingerspelling (see Results).

In Fig 2, self-rating scores of cognitive strategy during the maintenance stage are presented with respect to the sequence length and stimulus type. These self-rating measures revealed participants overall relied on the “phonological” strategy more greatly than on the “visuospatial” strategy during maintenance (t_12_ = 1.86, p < 0.05). For each participant, we further calculated the differences between the phonological and visuospatial strategies and submitted them to ANCOVA with a factor of stimulus type (letters and fingers) and a covariate of sequence length (4–7 syllables in length). This analysis revealed a significant effect of stimulus type (F [1, 12] = 19.8, p = 0.0008) and sequence length (F [1, 12] = 6.9, p = 0.02). The interaction between stimulus type and sequence length was non-significant (F [1, 12] = 0.8, p = 0.4). These findings suggest that (1) at the level of subjective introspection, participants utilized the phonological memory, rather than visuospatial memory, irrespective of the stimulus type, (2) this subjective reliance on phonological memory increased with sequence length and did not differ between letters and fingers.

### Brain imaging data

We first examined global neural activation during the encoding and decision stages. The encoding stage relative to the baseline produced distributed and left-predominant activation of the bilateral occipital, parietal, temporal and frontal regions. Bilateral occipital regions showed greater activation for fingers relative to letters, whereas no region showed greater activation for letters relative to fingers. Since finger stimuli were presented as moving images each having much more complex visual configurations and longer presentation durations than letter stimuli (see Materials and Methods), this activation difference in early visual areas can be attributed to these differences in physical features between letters and fingerspelling. On the other hand, the decision stage relative to the baseline activated the bilateral frontoparietal regions, including the precentral and postcentral gyri, with a trend of left hemispheric lateralization. No brain region showed significant activation difference between letters and fingers.

**Fig 3:**
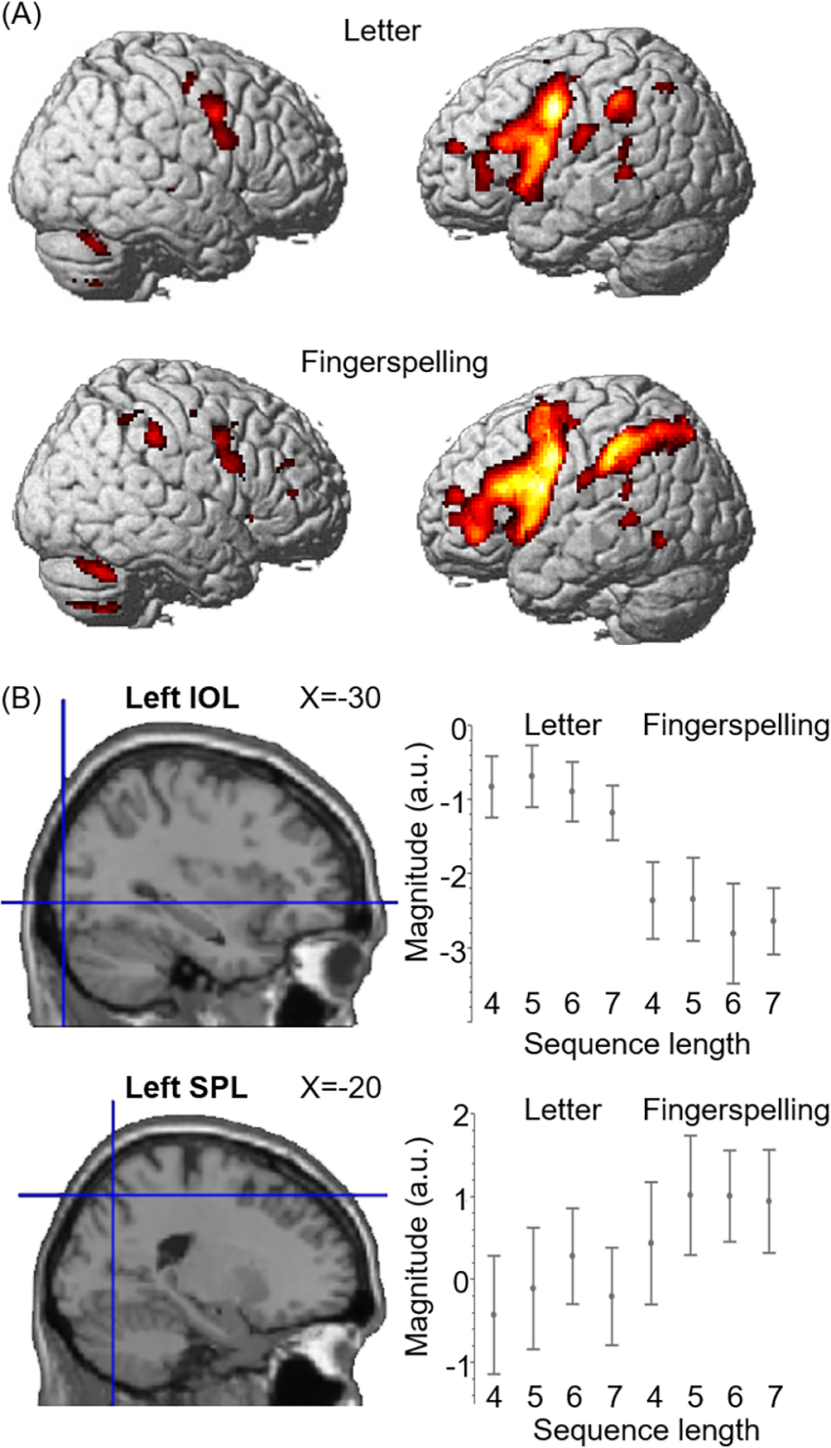
Overall pattern of activation during the maintenance stage. (A) Brain activation produced by letter and fingerspelling stimuli. The letter and finger condition both produced left-predominant activation in frontal-temporal regions. (B) Modality-specific activation during the maintenance stage. The left inferior occipital gyrus was more strongly activated for letters than for fingers (top). Conversely, the left superior parietal lobule was activated more greatly for fingers than for letters (bottom).

**Fig 4:**
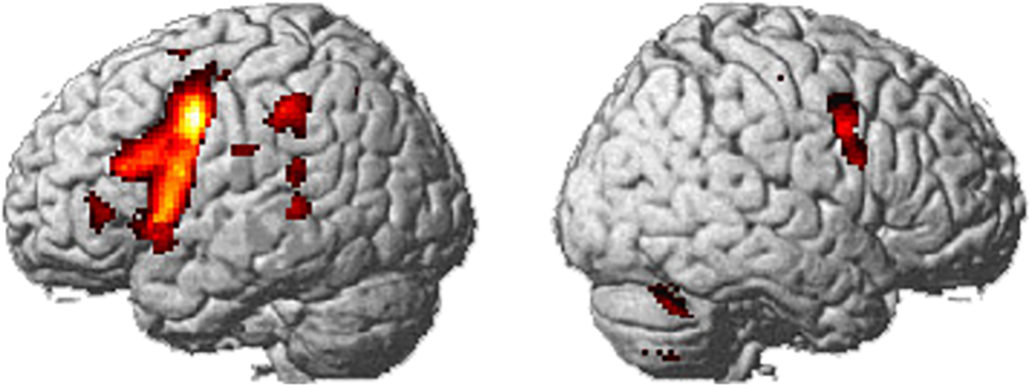
Global activation during the maintenance stage. Conjunction analysis of the letter and fingerspelling conditions revealed the left-hemisphere network including the perisylvian language area.

Next, we looked at neural activation during the maintenance stage more closely. The maintenance stage relative to the baseline produced bilateral and left-predominant activation in the precentral gyrus, left inferior frontal gyrus, left supplementary motor area, left superior temporal gyrus, left inferior parietal lobule. Letters and fingers both activated bilateral frontotemporal regions, including the superior temporal gyrus, supplementary motor area, precentral gyrus, opercular part of inferior frontal gyrus, relative to the baseline (Fig 3A). We then performed conjunction analysis to identify brain regions commonly activated by letters and fingers. This analysis revealed a distributed modality-independent network in the left hemisphere (Fig 4), including the precentral gyrus (-54, 0, 42, Z > 8), opercular part of inferior frontal gyrus (-52, 10, 6, Z > 8), supplementary motor area (-6, 6, 54, Z > 8), superior temporal gyrus (-58–38–4, Z = 7.18), inferior parietal lobule (-48, -38, 44, Z = 7.24).

We then assessed the effects of input modality and sequence length using an ANCOVA which included the stimulus type (letters and fingers) as a factor and sequence length (4–7 syllables) as a covariate. The effect of stimulus type was found in the bilateral inferior occipital gyri (IOG), which showed greater activation to letters than to fingers (Table 2). While these lateral occipital areas exhibited reduced activation relative to fixation (Fig 3B), this pattern of neural response (“deactivation”) is consistent with previous studies showing negative or only weak activation during phonological maintenance [19, 20]. By contrast, fingers activated the left inferior parietal lobule (IPL), superior parietal lobule (SPL), middle frontal gyrus and precuneus relative to letters (Table 2 and Fig 3B). This part of the middle frontal gyrus (-30, 4, 62) was located close to the previously reported coordinates of the dorsal premotor area (PMd, Z = 62, [21]), a brain region known to be involved in action planning, preparation and learning of arm with hand and finger [22–24]. Bilateral supplementary motor area showed increasingly greater activation with the sequence length of fingers (-10, 18, 36, Z = 5.70, and 2, 16, 52, Z = 5.10), while the opercular part of the left inferior frontal gyrus (IFG) showed increased activation with the sequence length of letters (-58, 14, 10, Z = 4.88). No brain region showed significant interaction between the factor of stimulus type and sequence length.

**Table 2:** Modality-specific activations during the maintenance stage.

Cluster size(mm^3^)	MNI coordinates			Z value	Side	Anatomical Region
	x	y	z			
*Fingerspelling > Letter*						
1688	-26	-2	46	6.33	L	Middle frontal cortex
	-30	4	62	5.91	L	Middle frontal cortex
3184	-36	-36	34	6.10	L	Inferior parietal lobule
	-38	-46	46	5.65	L	Inferior parietal lobule
	-36	-54	52	5.23	L	Inferior parietal lobule
1344	-8	-66	54	5.99	L	Precuneus
	-20	-70	52	5.23	L	Superior parietal lobule
*Letter > Fingerspelling*						
640	-32	-90	-4	5.72	L	Inferior occipital lobule
160	38	-84	16	5.61	R	Inferior occipital lobule

We further examined the possible contribution of three cortical regions, i.e., the left IOG that is known to play a key role in letter perception [25, 26], SPL previously associated with sign language processing and production [27–30], PMd associated with motor learning and planning [23, 24, 31] and IFG previously associated with lexical learning [16, 32]. To determine the possible role of these activations, we created 5-mm radius spherical regions of interest (ROIs) on IOG (-40, -83, -7) [26], SPL (-11, -67, 66) [30], PMd (-28, -10, 56) [24], IFG (-52, 9, 22) [16] (these MNI coordinates were obtained using a nonlinear spatial transformation of the original coordinates data in Talairach space [33]). For each region, the magnitude of activation relative to the baseline was evaluated using ANCOVA with a covariate of self-rating scale of cognitive strategy (differences in ‘phonological’ between ‘visuospatial’). This analysis revealed significant or marginally significant effects of strategy in the left IOG (F [1, 12] = 9.8, p = 0.009) and SPL (F [1, 12] = 5.1, p = 0.05), whereas the same effect did not reach significance either in the left PMd (F [1, 12] = 0.3 p = 0.6) or in the IFG (F [1, 12] = 1.0 p = 0.3). These findings suggest that the nature of cognitive strategy during maintenance exerts a significant impact on the neural activation of left IOG and SPL.

In addition, we examined the functional connectivity of the left IFG since this region is known to play a key role in the storage of verbal information [34, 35]. That is, we examined whether stimulus-modality (letters vs. fingers) changed the magnitude of functional coupling between this region and those occipitoparietal regions associated with letter perception and sign language processing. Indeed, this PPI analysis revealed stronger functional coupling (1) between the IFG and the left IOG (-32, -92, -6, Z = 2.58, p = 0.005) in the ‘Letter > Finger’ contrast and (2) between the IFG and the left SPL (-28, -38, 38, Z = 2.66, p = 0.004) in the ‘Finger > Letter’ contrast, respectively. These findings suggest that the left IOG specifically has a functional connectivity with the left IFG in letters, whereas the SPL specifically have a functional connectivity with the left IFG in fingers.

## Discussion

The present study investigated the neural correlates of verbal STM in sign interpreters while they learned novel words from letter and fingerspelling inputs. The sequential memory span task used for the present study is generally thought to engage the phonological STM irrespective of whether sensory inputs are presented as visual or auditory stimuli [20, 36, 37]. Indeed, the present behavioral analysis confirmed that participants subjectively relied on the phonological STM during the maintenance stage similarly for the letter and fingerspelling stimuli despite the radical difference in the nature of visual inputs.

The present fMRI results showed that letter and fingerspelling stimuli both produced largely overlapping and left-predominant activation in the inferior parietal lobule, superior temporal gyrus, inferior frontal gyrus, supplementary motor area and ventral premotor area. These brain regions are consistent with common activation areas previously associated with phonological STM for auditory and letter stimuli [20, 37, 38]. These regions are therefore likely to constitute a modality-independent neural circuitry for maintaining higher-order, abstract linguistic representations, which can be activated by fingerspelling inputs presented as handshape, location and movement.

On the other hand, we observed that letter stimuli activated the bilateral IOG more strongly than finger stimuli, whereas the opposite contrast revealed stronger activation of the left SPL. Moreover, PPI analysis revealed that the left IOG and SPL both showed stimulus-dependent changes in functional connectivity with the left IFG, i.e., a brain region associated with lexical retrieval and learning [16]. Since abstract representation of those visual stimuli, such as letter and fingerspelling, are generally thought to be stored in these posterior sensory regions [39–41], these findings can be interpreted as reflecting top-down activation signals arising from the left IFG during the effortful retrieval of visual and visuomotor memory stored in the occipitoparietal regions. This interpretation concurs with another finding that voluntary control of cognitive strategies (visuospatial or phonological) as measured with self-rating scales modulated the magnitude of neural activation in the IOG and SPL. More specifically, IOG and SPL activations are likely to reflect top-down amplification during the effortful retrieval of visual memory for letter-shapes and hand-shapes, respectively. This is in good accord with a previous study showing that written word learning depends on top-down amplification in which the left IFG provides semantic or phonological cues to lower-level areas including occipital cortex [42]. In addition, the present results from functional connectivity analysis imply that visuospatial memory, rather than motor memory, plays a main role in verbal learning via sign language, since only the SPL, but not the left PMd, showed significant changes in functional coupling with the left IFG. More generally, however, it should be noted that verbal STM in sign language probably involves not only these dorsal parietal regions but also other frontotemporal regions tightly interconnected with the IFG, such as the left superior temporal gyrus, premotor area, fusiform gyrus and supplementary motor area [43]. This is because our fMRI results only showed modality-specific (i.e., specific to letters or fingers) changes in functional connectivity with the left IFG, rather than more domain-general, modality-independent effects. We therefore propose that the left IFG exerts modality-specific connectivity with the IOG and SPL during verbal learning in hearing signers.

As described before, previous neuroimaging studies reported conflicting results as to whether the dorsal frontal-parietal pathway is involved in the verbal STM via sign language [6, 8]. That is, it remained unclear whether (1) verbal STM via sign language requires specific neural systems, such as the dorsal parietal cortex, or (2) it can be achieved by the same classical language network for spoken language processing since verbal STM is supposed to handle abstract linguistic representations independent of sensory modality. The existing neuroimaging literature on sign language seems unable to answer the question, because all those previous studies compared neural correlates of STM while hearing and deaf subjects were engaged in spoken and signed language processing, respectively. Obviously, however, these differences in participants and the nature of stimuli create a large confounding factor in assessing the dorsal parietal contribution during verbal learning via sign language inputs. To identify the possible modality-specific and modality-independent components of verbal STM via sign language, we used a within-participant design with proficient bilinguals of signed and spoken language. The present results revealed support the view for the dorsal parietal contribution during verbal learning in sign language, because we observed the strong dorsal parieto-frontal activation in the SPL and PMd specific to fingerspelling inputs and ventral occipito-frontal activation in the IOG and IFG specific to letter inputs.

Another interesting possibility is that the observed effects in the SPL with PMd during fingerspelling may reflect the activation of the human mirror neuron system that is thought to match a viewed act with an actual self-movement and allow us understand others’ action [29, 44]. In fact, the mirror neuron system has been shown to play a central role in the production of hand action, including both meaningful and meaningless gestures [5, 21, 45] and may be also involved in sign language processing [29]. Therefore, the observed fronto-parietal activation may reflect silent rehearsal using action imagery or imitation of sign language, mediated by mirror neuron system.

The present comparisons between letter and fingerspelling stimuli allowed us to roughly match the amount and pattern of attentional allocation between finger and non-finger stimuli (i.e., participants can be focused on visual stimuli on the screen and do not need to allocate attention to visual and auditory stimuli) and eliminate large differences in sensory neural activation which should arise when contrasting visual and auditory stimuli. Compared to spoken sound stimuli, our experimental paradigm with visual stimuli may be more suitable for exploring the neural basis of verbal STM in congenitally deaf people in future research. Obviously, however, the present design also suffered from some different types of confounding factors, such as the differences in stimulus shapes and durations between letters and fingers. Nevertheless, the potential contribution from those stimulus materials during encoding to the observed neural effects during maintenance should be minimal, because (1) the duration of the maintenance stage in itself was identical between letters and fingers (12 s) and far longer than the duration difference of the encoding stage (0.6 s to 1 s), (2) there was no significant difference of accuracy rate between the letter and finger memory task, suggesting there is not an extraordinary attention during the maintenance phase in the finger condition.

Additionally, it is important to take caution in generalizing the present findings to explain the neurocognitive mechanism in deaf patients, which should be addressed in future research with native sign users. Yet we think that the present imaging results shed light on the previously unknown aspects of neural functioning in late bilingual signers and thereby allow us to extend the existing knowledge about the neural basis of sign language.

## Conclusions

**Fig 5:**
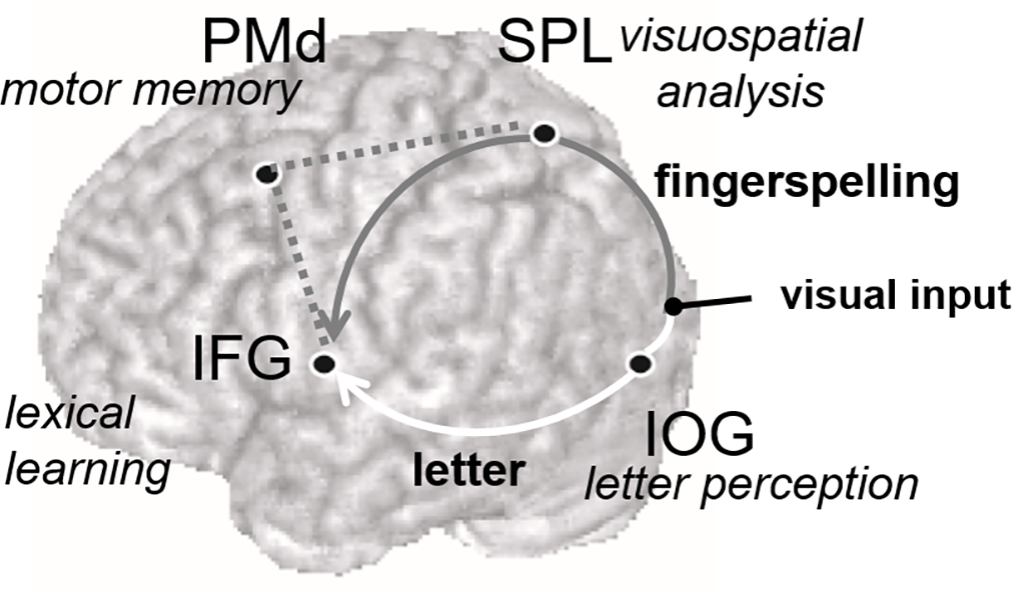
Modality-specific neural components for converting letter and fingerspelling inputs into phonological STM during verbal learning. The left IOG involved in letter perception is known to have fast and direct connections with the left IFG involved in speech production and lexical learning. This ventral occpitofrontal connection constitutes a specific neural pathway involved in verbal learning via letter inputs (white line). The dorsal parietofrontal connection linking the left SPL with the left IFG serves as a visuomotor pathway for verbal learning via sign language learning (gray line). The left PMd selectively activated by fingerspelling inputs is also known to have structural and functional coupling with the posterior parietal and inferior frontal regions [23] and thus likely to operate with this dorsal neural system (dotted line).

To summarize, the present results showed that a classical left-hemisphere language area is strongly activated even during verbal learning via fingerspelling. More importantly, our results also revealed two modality-specific neural connections operating for language learning (Fig 5). Namely, the ventral occipitofrontal connection between the left IOG and the left IFG is likely to constitute a specific neural pathway for verbal learning via letter inputs. In fact, the IFG has been thought to have direct connection with the lateral occipital region [46–49]. On the other hand, the dorsal parietofrontal connection linking the left SPL with the left IFG is likely to serve as a visuomotor pathway for verbal learning in sign language. These modality-specific systems each probably mediate top-down amplification of verbal and visuospatial memory during the effortful retrieval and maintenance of novel word learning. On the other hand, since our participants are hearing and non-native users of sign language, it still remains elusive whether or not the dorsal visuomotor pathway acts similarly during verbal learning in native signers, either hearing or deaf. However, our data of non-native hearing signers are also important for issues of differences in neural organization form early and late bilinguals. Further studies are required to determine how these and other neural components are functioning during novel word learning in with native hearing signers or deaf signers.

## References

[1] Bellugi U, Fischer S. A comparison of sign language and spoken language. Cognition. 1972;1(2):173–200.

[2] Hanson VL, Liberman IY, Shankweiler D. Linguistic coding by deaf children in relation to beginning reading success. Journal of experimental child psychology. 1984;37(2):378–93. 6726116 10.1016/0022-0965(84)90010-9

[3] Baddeley A, Gathercole S, Papagno C. The phonological loop as a language learning device. Psychological review. 1998;105(1):158–73. 9450375 10.1037/0033-295x.105.1.158

[4] Hickok G, Buchsbaum B, Humphries C, Muftuler T. Auditory–motor interaction revealed by fMRI: speech, music, and working memory in area Spt. Cognitive Neuroscience, Journal of. 2003;15(5):673–82. 10.1162/089892903322307393 12965041

[5] Newman AJ, Supalla T, Fernandez N, Newport EL, Bavelier D. Neural systems supporting linguistic structure, linguistic experience, and symbolic communication in sign language and gesture. Proceedings of the National Academy of Sciences. 2015;112(37):11684–9. 10.1073/pnas.1510527112 PMC4577150 26283352

[6] Bavelier D, Newman A, Mukherjee M, Hauser P, Kemeny S, Braun A, et al Encoding, rehearsal, and recall in signers and speakers: Shared network but differential engagement. Cerebral Cortex. 2008;18(10):2263–74. doi: 10.1093/cercor/bhm248 18245041 10.1093/cercor/bhm248 PMC2733310

[7] Rönnberg J, Rudner M, Ingvar M. Neural correlates of working memory for sign language. Cognitive Brain Research. 2004;20(2):165–82. doi: 10.1016/j.cogbrainres.2004.03.002 15183389 10.1016/j.cogbrainres.2004.03.002

[8] Buchsbaum B, Pickell B, Love T, Hatrak M, Bellugi U, Hickok G. Neural substrates for verbal working memory in deaf signers: fMRI study and lesion case report. Brain and language. 2005;95(2):265–72. doi: 10.1016/j.bandl.2005.01.009 16246734 10.1016/j.bandl.2005.01.009

[9] Sehyr ZS, Petrich J, Emmorey K. Fingerspelled and Printed Words Are Recoded into a Speech-based Code in Short-term Memory. J Deaf Stud Deaf Educ. 2016 Epub 2016/10/30. doi: 10.1093/deafed/enw068 . 27789552 10.1093/deafed/enw068

[10] Tanida Y, Ueno T, Ralph MAL, Saito S. The roles of long-term phonotactic and lexical prosodic knowledge in phonological short-term memory. Memory & cognition. 2014:1–20. 25388521 10.3758/s13421-014-0482-2

[11] Nakayama M, Tanida Y, Saito S. Long-term phonological knowledge supports serial ordering in working memory. Journal of experimental psychology Learning, memory, and cognition. 2015;41(5):1570–8. Epub 2015/03/03. doi: 10.1037/a0038825 . 25730304 10.1037/a0038825

[12] Amano S, Kondo T. Japanese NTT database series: lexical properties of Japanese, word-frequency (II) Tokyo: Sanseido 2000.

[13] Tamaoka K, Makioka S. Frequency of occurrence for units of phonemes, morae, and syllables appearing in a lexical corpus of a Japanese newspaper. Behavior Research Methods, Instruments, & Computers. 2004;36(3):531–47. 10.3758/bf03195600 15641442

[14] Wilson M, Emmorey K. A visuospatial “phonological loop” in working memory: Evidence from American Sign Language. Memory & Cognition. 1997;25(3):313–20. 9184483 10.3758/bf03211287

[15] Xu J, Moeller S, Auerbach EJ, Strupp J, Smith SM, Feinberg DA, et al Evaluation of slice accelerations using multiband echo planar imaging at 3T. Neuroimage. 2013;83:991–1001. doi: 10.1016/j.neuroimage.2013.07.055 23899722 10.1016/j.neuroimage.2013.07.055 PMC3815955

[16] Raboyeau G, Marcotte K, Adrover-Roig D, Ansaldo AI. Brain activation and lexical learning: the impact of learning phase and word type. Neuroimage. 2010;49(3):2850–61. Epub 2009/10/20. doi: 10.1016/j.neuroimage.2009.10.007 . 19837173 10.1016/j.neuroimage.2009.10.007

[17] Friston KJ, Buechel C, Fink GR, Morris J, Rolls E, Dolan RJ. Psychophysiological and modulatory interactions in neuroimaging. Neuroimage. 1997;6(3):218–29. Epub 1997/11/05. doi: 10.1006/nimg.1997.0291 . 9344826 10.1006/nimg.1997.0291

[18] Tzourio-Mazoyer N, Landeau B, Papathanassiou D, Crivello F, Etard O, Delcroix N, et al Automated anatomical labeling of activations in SPM using a macroscopic anatomical parcellation of the MNI MRI single-subject brain. Neuroimage. 2002;15(1):273–89. doi: 10.1006/nimg.2001.0978 11771995 10.1006/nimg.2001.0978

[19] Laurienti PJ, Burdette JH, Wallace MT, Yen Y-F, Field AS, Stein BE. Deactivation of sensory-specific cortex by cross-modal stimuli. Journal of Cognitive Neuroscience. 2002;14(3):420–9. doi: 10.1162/089892902317361930 11970801 10.1162/089892902317361930

[20] Crottaz-Herbette S, Anagnoson R, Menon V. Modality effects in verbal working memory: differential prefrontal and parietal responses to auditory and visual stimuli. Neuroimage. 2004;21(1):340–51. 14741672 10.1016/j.neuroimage.2003.09.019

[21] Schubotz RI, von Cramon DY. Functional–anatomical concepts of human premotor cortex: evidence from fMRI and PET studies. Neuroimage. 2003;20:S120–S31. 14597305 10.1016/j.neuroimage.2003.09.014

[22] Buccino G, Binkofski F, Fink GR, Fadiga L, Fogassi L, Gallese V, et al Action observation activates premotor and parietal areas in a somatotopic manner: an fMRI study. The European journal of neuroscience. 2001;13(2):400–4. Epub 2001/02/13. . 11168545

[23] Hoshi E, Tanji J. Distinctions between dorsal and ventral premotor areas: anatomical connectivity and functional properties. Current opinion in neurobiology. 2007;17(2):234–42. Epub 2007/02/24. doi: 10.1016/j.conb.2007.02.003 . 17317152 10.1016/j.conb.2007.02.003

[24] Amiez C, Hadj-Bouziane F, Petrides M. Response selection versus feedback analysis in conditional visuo-motor learning. Neuroimage. 2012;59(4):3723–35. Epub 2011/11/02. doi: 10.1016/j.neuroimage.2011.10.058 . 22040737 10.1016/j.neuroimage.2011.10.058

[25] Ellis AW, Ferreira R, Cathles-Hagan P, Holt K, Jarvis L, Barca L. Word learning and the cerebral hemispheres: from serial to parallel processing of written words. Philosophical transactions of the Royal Society of London Series B, Biological sciences. 2009;364(1536):3675–96. Epub 2009/11/26. doi: 10.1098/rstb.2009.0187 ; PubMed Central PMCID: PMCPMC2846318. 19933140 10.1098/rstb.2009.0187 PMC2846318

[26] Cai Q, Paulignan Y, Brysbaert M, Ibarrola D, Nazir TA. The left ventral occipito-temporal response to words depends on language lateralization but not on visual familiarity. Cereb Cortex. 2010;20(5):1153–63. Epub 2009/08/18. doi: 10.1093/cercor/bhp175 . 19684250 10.1093/cercor/bhp175

[27] MacSweeney M, Woll B, Campbell R, Calvert GA, McGuire PK, David AS, et al Neural correlates of British sign language comprehension: spatial processing demands of topographic language. J Cogn Neurosci. 2002;14(7):1064–75. Epub 2002/11/07. doi: 10.1162/089892902320474517 . 12419129 10.1162/089892902320474517

[28] Emmorey K, Grabowski T, McCullough S, Ponto LL, Hichwa RD, Damasio H. The neural correlates of spatial language in English and American Sign Language: a PET study with hearing bilinguals. Neuroimage. 2005;24(3):832–40. Epub 2005/01/18. doi: 10.1016/j.neuroimage.2004.10.008 . 15652318 10.1016/j.neuroimage.2004.10.008

[29] Corina DP, Knapp H. Sign language processing and the mirror neuron system. Cortex. 2006;42(4):529–39. 16881265 10.1016/s0010-9452(08)70393-9

[30] Emmorey K, Mehta S, Grabowski TJ. The neural correlates of sign versus word production. Neuroimage. 2007;36(1):202–8. doi: 10.1016/j.neuroimage.2007.02.040 17407824 10.1016/j.neuroimage.2007.02.040 PMC1987366

[31] Levanen S, Uutela K, Salenius S, Hari R. Cortical representation of sign language: comparison of deaf signers and hearing non-signers. Cereb Cortex. 2001;11(6):506–12. Epub 2001/05/29. . 11375912 10.1093/cercor/11.6.506

[32] Price CJ, Devlin JT. The interactive account of ventral occipitotemporal contributions to reading. Trends in cognitive sciences. 2011;15(6):246–53. doi: 10.1016/j.tics.2011.04.001 21549634 10.1016/j.tics.2011.04.001 PMC3223525

[33] Lacadie CM, Fulbright RK, Rajeevan N, Constable RT, Papademetris X. More accurate Talairach coordinates for neuroimaging using non-linear registration. Neuroimage. 2008;42(2):717–25. doi: 10.1016/j.neuroimage.2008.04.240 18572418 10.1016/j.neuroimage.2008.04.240 PMC2603575

[34] Owen AM. The role of the lateral frontal cortex in mnemonic processing: the contribution of functional neuroimaging. Experimental brain research. 2000;133(1):33–43. Epub 2000/08/10. doi: 10.1007/s002210000398 . 10933208 10.1007/s002210000398

[35] Wager TD, Smith EE. Neuroimaging studies of working memory. Cognitive, Affective, & Behavioral Neuroscience. 2003;3(4):255–74. 10.3758/cabn.3.4.255 15040547

[36] Schumacher EH, Lauber E, Awh E, Jonides J, Smith EE, Koeppe RA. PET evidence for an amodal verbal working memory system. Neuroimage. 1996;3(2):79–88. doi: 10.1006/nimg.1996.0009 9345478 10.1006/nimg.1996.0009

[37] Ruchkin DS, Berndt RS, Johnson R Jr, Ritter W, Grafman J, Canoune HL. Modality-specific processing streams in verbal working memory: evidence from spatio-temporal patterns of brain activity. Cognitive Brain Research. 1997;6(2):95–113. 9450603 10.1016/s0926-6410(97)00021-9

[38] Montgomery KJ, Isenberg N, Haxby JV. Communicative hand gestures and object-directed hand movements activated the mirror neuron system. Social cognitive and affective neuroscience. 2007;2(2):114–22. doi: 10.1093/scan/nsm004 18985130 10.1093/scan/nsm004 PMC2555455

[39] Harrison SA, Tong F. Decoding reveals the contents of visual working memory in early visual areas. Nature. 2009;458(7238):632–5. Epub 2009/02/20. doi: 10.1038/nature07832 ; PubMed Central PMCID: PMCPMC2709809. 19225460 10.1038/nature07832 PMC2709809

[40] Serences JT, Ester EF, Vogel EK, Awh E. Stimulus-specific delay activity in human primary visual cortex. Psychol Sci. 2009;20(2):207–14. Epub 2009/01/28. doi: 10.1111/j.1467-9280.2009.02276.x ; PubMed Central PMCID: PMCPMC2875116. 19170936 10.1111/j.1467-9280.2009.02276.x PMC2875116

[41] Christophel TB, Cichy RM, Hebart MN, Haynes J-D. Parietal and early visual cortices encode working memory content across mental transformations. Neuroimage. 2015;106:198–206. doi: 10.1016/j.neuroimage.2014.11.018 25463456 10.1016/j.neuroimage.2014.11.018

[42] Blasi V, Young AC, Tansy AP, Petersen SE, Snyder AZ, Corbetta M. Word retrieval learning modulates right frontal cortex in patients with left frontal damage. Neuron. 2002;36(1):159–70. Epub 2002/10/09. . 12367514 10.1016/s0896-6273(02)00936-4

[43] Price CJ. A review and synthesis of the first 20years of PET and fMRI studies of heard speech, spoken language and reading. Neuroimage. 2012;62(2):816–47. doi: 10.1016/j.neuroimage.2012.04.062 22584224 10.1016/j.neuroimage.2012.04.062 PMC3398395

[44] Rizzolatti G, Fogassi L, Gallese V. Motor and cognitive functions of the ventral premotor cortex. Current opinion in neurobiology. 2002;12(2):149–54. 12015230 10.1016/s0959-4388(02)00308-2

[45] Tanaka S, Inui T, Iwaki S, Konishi J, Nakai T. Neural substrates involved in imitating finger configurations: an fMRI study. Neuroreport. 2001;12(6):1171–4. 11338186 10.1097/00001756-200105080-00024

[46] Jobard G, Crivello F, Tzourio-Mazoyer N. Evaluation of the dual route theory of reading: a metanalysis of 35 neuroimaging studies. Neuroimage. 2003;20(2):693–712. Epub 2003/10/22. doi: 10.1016/S1053-8119(03)00343-4 . 14568445 10.1016/S1053-8119(03)00343-4

[47] Cornelissen PL, Kringelbach ML, Ellis AW, Whitney C, Holliday IE, Hansen PC. Activation of the left inferior frontal gyrus in the first 200 ms of reading: evidence from magnetoencephalography (MEG). PloS one. 2009;4(4):e5359 Epub 2009/04/28. doi: 10.1371/journal.pone.0005359 ; PubMed Central PMCID: PMCPMC2671164. 19396362 10.1371/journal.pone.0005359 PMC2671164

[48] Wheat KL, Cornelissen PL, Frost SJ, Hansen PC. During visual word recognition, phonology is accessed within 100 ms and may be mediated by a speech production code: evidence from magnetoencephalography. The Journal of neuroscience: the official journal of the Society for Neuroscience. 2010;30(15):5229–33. Epub 2010/04/16. doi: 10.1523/jneurosci.4448-09.2010 ; PubMed Central PMCID: PMCPMC3419470. 20392945 10.1523/JNEUROSCI.4448-09.2010 PMC3419470

[49] Klein M, Grainger J, Wheat KL, Millman RE, Simpson MI, Hansen PC, et al Early Activity in Broca's Area During Reading Reflects Fast Access to Articulatory Codes From Print. Cereb Cortex. 2015;25(7):1715–23. Epub 2014/01/23. doi: 10.1093/cercor/bht350 . 24448559 10.1093/cercor/bht350

